# Associations Between Pain Reactivity to Worse Sleep and Health Outcomes

**DOI:** 10.1093/geroni/igaf122.3545

**Published:** 2025-12-31

**Authors:** Christina Mu, Brent Small, Christina McCrae, Lindsay Peterson, Ross Andel, Katie Stone, Soomi Lee

**Affiliations:** University of California, San Francisco, University of California, San Francisco, California, United States; University of North Carolina at Chapel Hill, Chapel Hill, North Carolina, United States; University of South Florida, Tampa, Florida, United States; University of South Florida, Tampa, Florida, United States; Arizona State University, Arizona State University, Arizona, United States; California Pacific Medical Center Research Institute, Castro Valley, California, United States; The Pennsylvania State University, University Park, Pennsylvania, United States

## Abstract

**Objectives:**

Sleep and pain are co-occurring issues in adulthood. Pain reactivity to worse sleep refers to person-specific changes in daily pain following nights of shorter-than-usual sleep duration or poorer-than-usual sleep quality. We examined the cross-sectional and longitudinal associations of sleep-related pain reactivity with psychological distress and the number of chronic conditions.

**Methods:**

Data were obtained from the Work, Family, and Health Study, which collected data from healthcare and information technology workers (n = 311; Mage=41.38 years), who completed 8-days of daily diary. We controlled for sociodemographic and health covariates in a series of multilevel structural equation models and growth curve models in Mplus. Sensitivity analyses adjusted for sleep and pain medications and stratified analyses by age.

**Results:**

Those who reported more pain locations and severity following poorer-than-usual sleep quality had more concurrent distress (higher pain location reactivity to worse sleep quality: B = 1.03, p<.001; higher pain severity reactivity to worse sleep quality: B = 0.56, p=.001). In the growth curve models, higher pain location reactivity to poorer sleep quality was associated with higher concurrent distress (Path Est.=1.67, p=.023) and decreases in distress over time (Path Est.=-0.74, p=.032). Higher pain severity reactivity to poorer sleep quality was associated with higher concurrent distress (Path Est.=0.62, p=.030) but not changes in distress over time. Pain reactivity to worse sleep was not associated with concurrent or change in the number of chronic conditions.

**Discussion:**

Future directions are to examine the influence of pain reactivity to worse sleep in larger and more diverse samples across longer follow-up periods.

